# The first late cretaceous mawsoniid coelacanth (Sarcopterygii: Actinistia) from North America: Evidence of a lineage of extinct ‘living fossils’

**DOI:** 10.1371/journal.pone.0259292

**Published:** 2021-11-11

**Authors:** Lionel Cavin, Pablo Toriño, Nathan Van Vranken, Bradley Carter, Michael J. Polcyn, Dale Winkler

**Affiliations:** 1 Department of Geology and Palaeontology, Natural History Museum, Geneva, Switzerland; 2 Instituto de Ciencias Geológicas, Facultad de Ciencias, UdelaR, Montevideo, Uruguay; 3 STEM Division, Potomac State College, Keyser, West Virginia, United States of America; 4 4 Independent Researcher, 420 Kennedy Drive, Crowley, TX, United States of America; 5 Huffington Department of Earth Sciences, Southern Methodist University, Dallas, TX, United States of America; Università degli Studi di Torino, ITALY

## Abstract

Today, the only living genus of coelacanth, *Latimeria* is represented by two species along the eastern coast of Africa and in Indonesia. This sarcopterygian fish is nicknamed a "living fossil", in particular because of its slow evolution. The large geographical distribution of *Latimeria* may be a reason for the great resilience to extinction of this lineage, but the lack of fossil records for this genus prevents us from testing this hypothesis. Here we describe isolated bones (right angular, incomplete basisphenoid, fragments of parasphenoid and pterygoid) found in the Cenomanian Woodbine Formation in northeast Texas that are referred to the mawsoniid coelacanth *Mawsonia* sp. In order to assess the impact of this discovery on the alleged characteristic of "living fossils" in general and of coelacanths in particular: 1) we compared the average time duration of genera of ray-finned fish and coelacanth in the fossil record; 2) we compared the biogeographic signal from *Mawsonia* with the signal from the rest of the vertebrate assemblage of the Woodbine formation; and 3) we compared these life traits with those of *Latimeria*. The stratigraphical range of *Mawsonia* is at least 50 million years. Since *Mawsonia* was a fresh, brackish water fish with probably a low ability to cross large sea barriers and because most of the continental components of the Woodbine Fm vertebrate assemblage exhibit Laurasian affinities, it is proposed that the *Mawsonia*’s occurrence in North America is more likely the result of a vicariant event linked to the break-up of Pangea rather than the result of a dispersal from Gondwana. The link between a wide geographic distribution and the resilience to extinction demonstrated here for *Mawsonia* is a clue that a similar situation existed for *Latimeria*, which allowed this genus to live for tens of millions of years.

## Introduction

The extant coelacanth, *Latimeria* is the only living genus of a lineage that split from other sarcopterygians over 400 million years ago. The two currently recognized species, *L*. *chalumnae* in the western Indian Ocean and *L*. *menadoensis* in Indonesia have low genetic diversity, and their genetic evolution rate is slower than that of other vertebrates indicated by their mitochondrial genome [1–3] as well as their nuclear genome [4], at least for the protein-coding genes [5, 6]. Although showing only minor morphological difference, the two extant species diverged 30 to 40 million years ago [7, 8]. The recent study of a specimen of *L*. *menadoensis* captured in eastern Indonesia indicates a molecular divergence with others populations of the same species 13 million years ago [3], possibly indicating the existence of a third distinct species of *Latimeria*. Observation in vivo and genetic studies indicate that *Latimeria* is a sedentary fish [9]. These evolutionary traits suggest that the lineage of *Latimeria* has evolved slowly over a large geographic area. This last trait may explain the great resilience to extinction of this lineage, since a correlation between geographic area and survival during mass and background extinctions has been demonstrated for several groups of invertebrates, e.g. Paleozoic brachiopods [10], marine molluscs [11, 12], planktonic foraminifera [13] and ostracods [14]. It should be noted that in terrestrial environments a different pattern has been observed for vertebrates. As with marine invertebrates, there is good extinction resilience for clades with larger geographic ranges than for those with smaller ranges throughout the Triassic and Jurassic, but this relationship is no longer observed at the end-Triassic mass extinction [15].

The complete absence of a Cenozoic fossil record of the *Latimeria* lineage, and indeed of any coelacanth, however, prevents the reconstruction of its evolutionary history, and the ability to test its supposedly slow rate of morphological evolution and its past geographic distribution. In the Cretaceous, the fossil record of the coelacanths is richer, although not abundant. Alongside the latimeriid coelacanths, a second family, the mawsoniids, thrived mainly in brackish and fresh water. They occupied a large geographic area covering all of Western Gondwana (South America, Africa and Madagascar) [16–25], with an incursion during the Late Cretaceous into Europe [26–28]. The systematics of the Cretaceous mawsoniids is still uncertain, particularly at the specific level, but it is increasingly clear that two sister genera, well supported by all cladistics analyses (e.g. [29, 30]), shared their stratigraphic range throughout the Cretaceous: *Mawsonia* occurred in the Late Jurassic and its range extended until the early Late Cretaceous, while *Axelrodichthys* is known from the late Early Cretaceous (although the ghost range of its lineage is expected to extend to the Late Jurassic if it is a sister of *Mawsonia*) and became extinct in the Late Cretaceous on the basis of our current knowledge [28].

The record of fossil coelacanths in the Cretaceous of Texas is extremely sparse and poorly understood. Thurmond [31] described fish material found in microfaunas from the Early Cretaceous Trinity Group of central Texas, including fragments that he tentatively referred as “?coelacanth.” The fragmentary specimens include jaws, and possible vomers, recovered from four localities (Lewis, Walnut Creek, Priddy and Weatherford). Winkler and Murry [32] reported possible coelacanth specimens found at the Paluxy Church locality, also in the Trinity Group. The Trinity Group is latest Aptian to Early Albian in age. Graf [33] described a small partial skull of a coelacanth as *Reidus hilli* from the basal Upper Albian Duck Creek Formation.

Here we describe new fossils of the first Cretaceous North American mawsoniid coelacanth. These specimens were recovered by one of us (BC) from two localities in the Woodbine Group: SMU locality v522, along the north edge of Lewisville Lake, Denton County, and at SMU locality v574, in Arlington, Tarrant County, Texas. Coelacanth fossils have not previously been reported from the Woodbine Group, which is Cenomanian in age, and therefore this represents the geologically youngest occurrence of a coelacanth in Texas. Other North American Cenomanian faunas from the Western Interior Seaway have produced abundant chondrichthyans and bony fishes, but no coelacanths [34–36]. Rare latimeriid coelacanths are known from younger Cretaceous faunas in North America [37].

## Material and methods

In this paper, we identify the new coelacanth material and investigate the palaeogeographical signal of this discovery alongside the rest of the vertebrate assemblage from the Woodbine Formation. We tackle evolutionary traits of the coelacanth lineage by comparing stratigraphical genus duration within the ray-finned fish clade (Actinopterygii) and coelacanth clade (Actinistia) in the Devonian–Palaeogene time interval. Based on the database of Cavin et al. [38], gathering approximately all known genera of coelacanths and ray-finned fish (Actinopterygii) ranging from the Devonian to the Paleocene, i.e. 58 and 1108 genera, respectively, we calculated the average time duration of genera, or genera survivorship, for each clade. We also calculated these parameters by distinguishing paleoenvironments in which the genera lived, i.e. marine on one side and fresh water plus brackish on the other side. We eventually discuss potential factors that may cause the peculiar evolutionary traits of the genera *Mawsonia* and *Latimeria*, i.e. their long stratigraphical range and wide geographical distribution.

The specimens were scanned in the Digital Earth Science Lab at SMU using a NextEngine HD scanner and Scan Studio software [39]. Scan processing was performed in Meshlab [40] to reduce polygon count. Scans were rendered for figures using Lightwave 2020 [41].

## Results

### Localities and geological setting (Fig 1)

**Fig 1:**
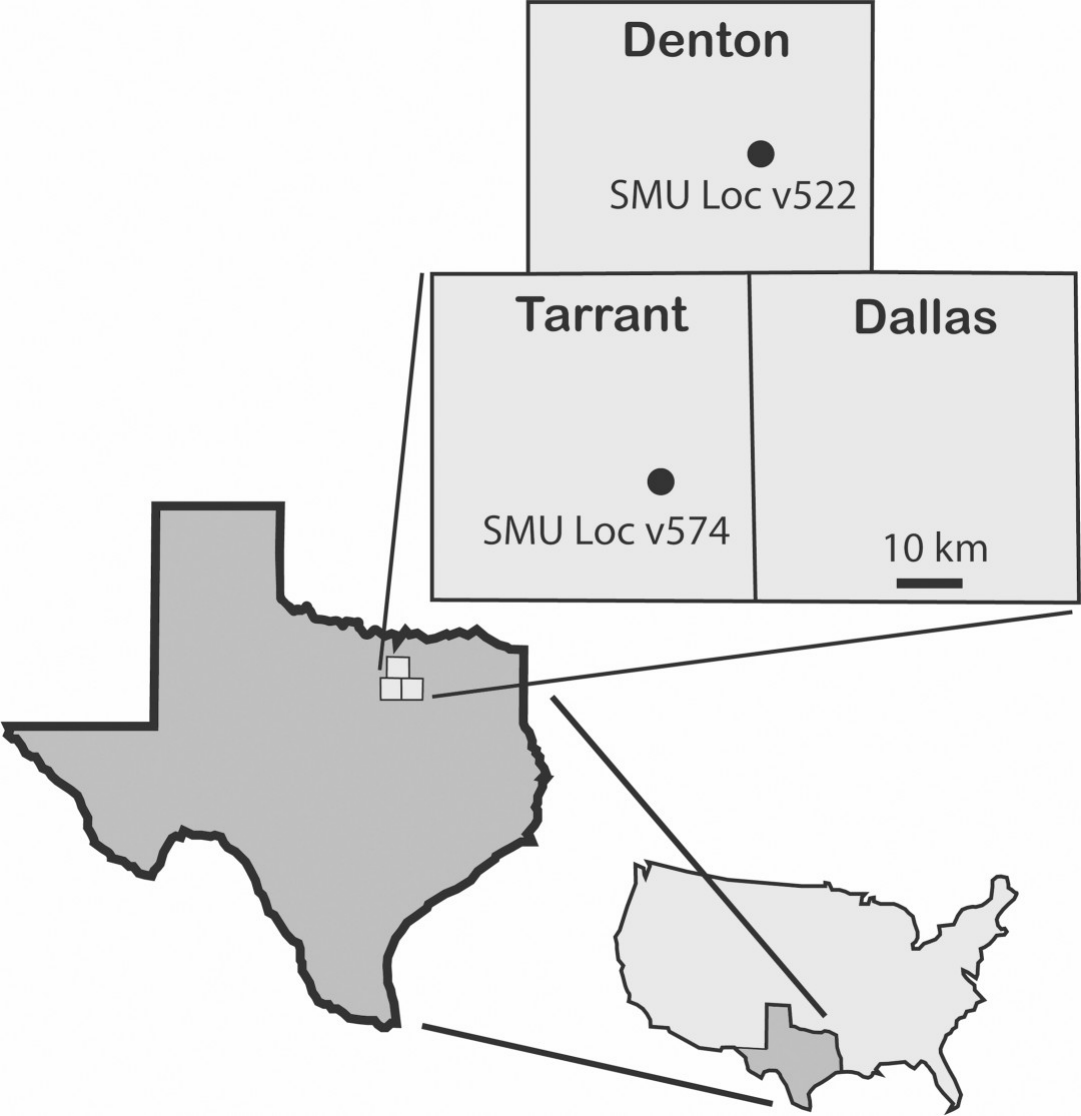
Map with the two localities (SMU Loc v522, SMU Loc v574) in the counties of north central Texas that have yielded remains of *Mawsonia* sp.

The Woodbine Group crops out along the Red River at the Texas-Oklahoma border and extends to the south and southwest in a narrow band just through the Dallas-Fort Worth Metroplex. In the subsurface, this sedimentary package thickens toward the East Texas Basin and has long played a major role in the oil and gas business [42–44]. Clastic sediments of the Woodbine Group were shed to the south and southwest from the Ouachita Mountains of Oklahoma and Arkansas [42, 45] during a major regression after the initial transgressive development of the Western Interior Seaway of North America [46, 47]. A temporary land connection was established between eastern (Appalachia) and western North America at this time, before being separated again until the end of the Cretaceous by transgression of the Western Interior Seaway [47–49]. Woodbine Group facies represent fluvial, deltaic, near-shore marine, and prodeltaic marine environments [42, 44, 45, 50].

In the vicinity of Dallas and Fort Worth, the Woodbine Group is stratigraphically bounded at the base by an unconformity on either the Grayson or Buda formations of the Comanche Series. These units are Early Cenomanian in age [51, 52]. Dodge [53] divided the outcropping Woodbine Formation into four members (Formations according to some) in the Dallas-Fort Worth area, but different schemes are used to the north along the Red River and in the subsurface [45, 54–58]. Moreman [59, 60] defined the Tarrant Formation in the Dallas-Fort Worth area, as the basal unit of the Eagle Ford Group overlying the Woodbine Group (see [61–63]). The Tarrant Formation stratigraphically overlies the Woodbine Group localities described here. Stephenson [64] and Denne et al. [56], however, advocate transferring the Tarrant Formation to their upper (Lewisville) formation of the Woodbine Group (see [65]). Others do not recognize the unit or include it in the Lewisville or Arlington Formation [53, 55, 66]. Regardless of assignment, the Tarrant Formation (as defined by Moreman [59, 60]) locally contains the ammonite *Conlinoceras tarrantense* (in contrast to typical Woodbine Group sediments below it), an early Middle Cenomanian marker [65], and therefore the localities of concern in this paper lie in the late Early Cenomanian. Sediments of the Woodbine Group grade southward and in the subsurface into fine grained marine sediments (Pepper Formation).

Paleogeographic models and paleoclimatic models during the Albian-Cenomanian part of the Cretaceous demonstrate a semi-arid to tropical belt that extends from the Gulf of Mexico into North Africa, Southern Europe, and northern South America [67, 68]. Clay mineralogy indicates an increase in precipitation from the Albian into the Cenomanian in north central Texas, and a warm sub-humid climate during deposition of the Woodbine Group [69].

Both marine invertebrates and vertebrates as well as fully terrestrial fauna and flora are known from the Woodbine Group [64, 70–72]. Of the vertebrate fauna, sharks and rays were historically the most extensively studied group [73–80]. Remains of the actinopterygians *Lepidotes* sp., pycnodonts and *Enchodus* sp. have been noted [81], but only a few fishes have been formally described, a lungfish and an ichthyodectiform teleost [82, 83]. A new pachyrhizodontid was named from the overlying Tarrant Formation [84]. As many as four taxa of turtles have been recorded from the Woodbine [85, 86], and diverse crocodylomorphs have been described recently [87–91]. Abundant tracks and remains of dinosaurs are known from the Woodbine, including ornithopods (*Protohadros byrdi*), nodosaurs, and theropods [87, 92–95]. Other tetrapods include an enantiornithine bird [96], and mammals [94, 97, 98].

All but one of the mawsoniid specimens described here come from Locality SMU v522, not far below the top of the Woodbine Group (referred to the Arlington “Member” by [82]). As in most places, the Woodbine Group strata in the area of SMU Loc. v522 occur in discontinuous outcrops and demonstrate great lateral facies variability [50]. Both marine and fully terrestrial vertebrates are found within the strata at the site. Hacker and Shimada [82] named a new fish, *Bardackichthys carteri*, from this locality. Another presumably marine taxon, the crocodylomorph *Terminonaris* cf *T*. *robusta* was discovered at another locality nearby [89]. Bones of the ornithopod *Protohadros* sp. are abundant here. Marine and terrestrial fossils also occur at the Arlington locality (v574), which lies low in the Woodbine Group in facies typical of the Dexter Formation. In addition to the basisphenoid described below, specimens from Locality v574 include bivalve molluscs, decapod crustaceans, crocodyliforms, and cf. *Protohadros*.

All necessary permits were obtained for the described study, which complied with all relevant regulations.

### Systematic paleontology

Actinistia Cope, 1871.
  Latimerioidei Schultze, 1993.
  Mawsoniidae Schultze, 1993.
  Genus *Mawsonia* Woodward in Mawson and Woodward, 1907
  *Mawsonia* sp.

#### Left angular (SMU 77688) (Fig 2)

**Fig 2:**
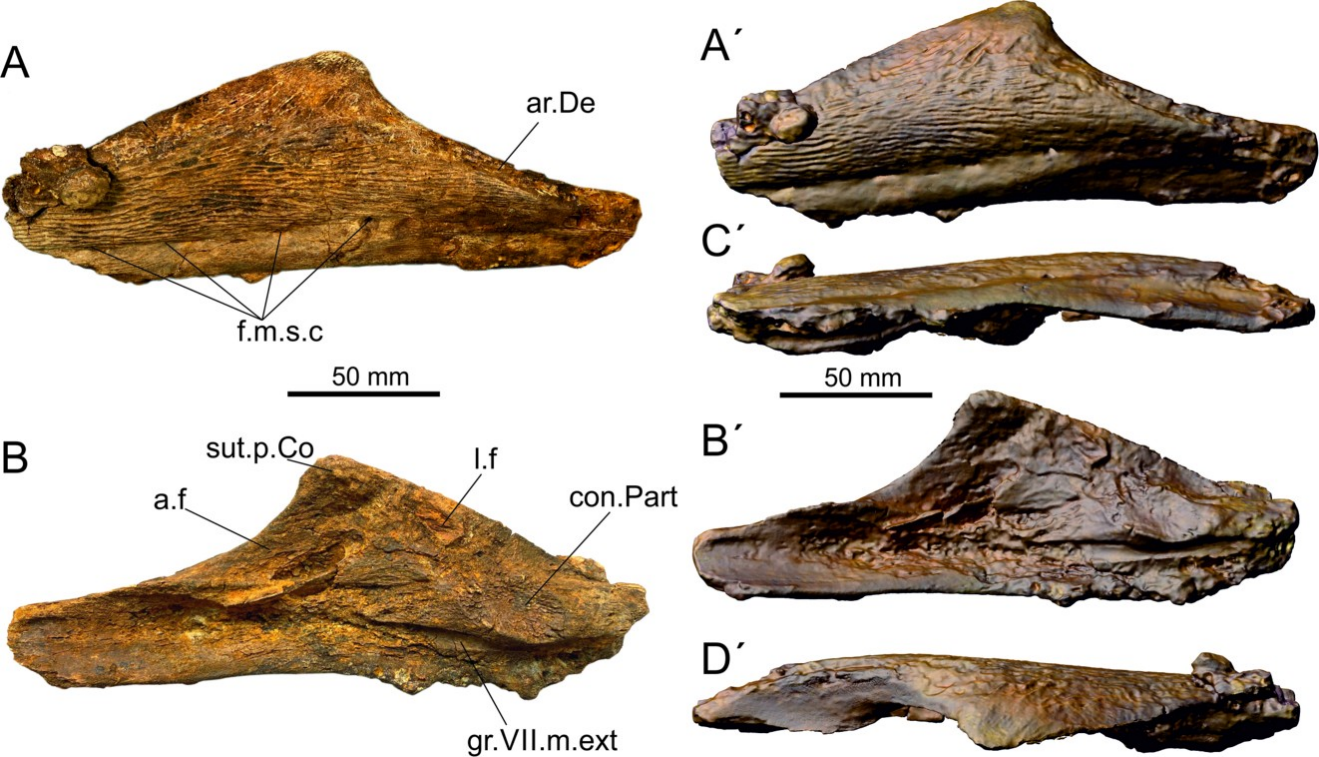
Photograph and surface rendering (apostrophes) of left angular (SMU 77688) of *Mawsonia* sp. from the Woodbine Formation in lateral (A), medial (B), ventral (C) and dorsal (D) views. Abbreviations: a.f, adductor fossa; ar.De: articular surface for dentary; con.Part, contact surface with prearticular; f.m.s.c, openings of the mandibular sensory canal; gr.VII.m.ext groove for external mandibular ramus of VII; l.f, longitudinal fossa; sut.p.Co, sutural contact surface with principal coronoid.

**Fig 3:**
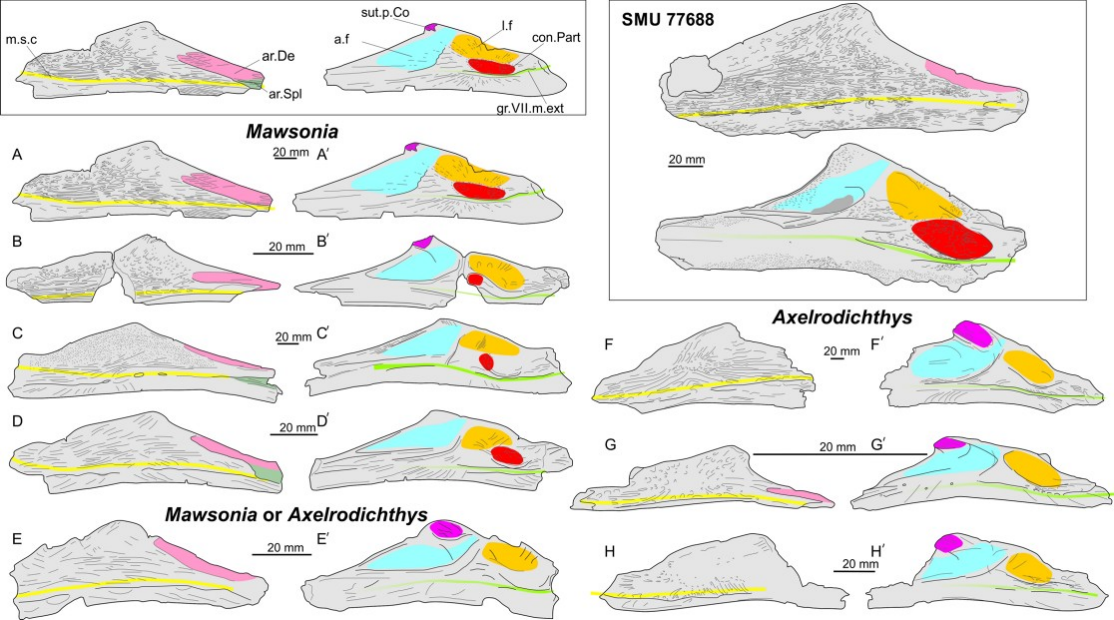
Comparison of the angular of the Woodbine Fm (upper right frame) with *Mawsonia* specimens (A-D) and *Axelrodichthys* specimens (F-H), and *Mawsonia* or *Axelrodichthys* in labial (A-H) and lingual (A’-H’) views. Left small frame, anatomical features. Abbreviations: a.f, aductor fossa (blue); ar.De: articular surface for dentary (pink); ar.Spl: articular surface for splenial (grey); con.Part, contact surface with prearticular (red); gr.VII.m.ext groove for external mandibular ramus of VII (green); l.f, longitudinal fossa (orange); m.s.c, mandibular sensory canal (yellow); sut.p.Co, sutural contact surface with principal coronoid (purple).

*Remark about the fossae on the angular of Mawsonia (Fig 3, box on the left).* In mawsoniids, the lingual face of the angular is hollowed out dorsally by two fossae separated by a ridge. Usually, the anterior fossa is called the adductor fossa, and the posterior fossa is called the Meckelian fossa. The latter was so likely named after Tabaste [18] who compared the situation of "*Mawsonia*" *lavocati* to *Latimeria chalumnae* and noticed that this pit was covered with Meckel’s cartilage in the extant species ("Elle est recouverte par le cartilage de Meckel qui, à cet endroit, forme la cavité d’articulation avec le palatocarré”). In fact, in an articulated lower jaw of a coelacanth, this depression contained the retroarticular and articular bones, both ossified from the Meckel cartilage and wedged between the angular laterally and the prearticular internally, and forming the glenoid cavity for the quadrate. This fossa is called here the longitudinal fossa following the expression used by Millot & Anthony [99]. Tabaste’s adductor fossa [18] is the space between the articular and the prearticular, i.e. the Meckelian fossa, partially filled with adductor muscles of the mandible [99, 100]. The adductor mandible muscle enter the cavity through an opening anterior to the articular and posterior the principal coronoid. The ridge on the principal coronoid present in most coelacanths leads the muscle fibers of the adductor mandibulae muscles. In *Mawsonia* and *Axelrodichthys*, the situation is a little different since the muscle fibers are enclosed anteriorly by the bony bridge formed by the principal coronoid. This bridge is formed by the corono-angular ligament in *Latimeria* [99]. Therefore, there is no clear separation between the Meckelian fossa and the adductor fossa. This interpretation corresponds to the interpretation of Forey [29].

A, *Mawsonia gigas* (BMNH P.10360) from the ?Late Hauterivian Marfim Fm., Brazil (redrawn inverted from Forey, 1998 (A) and Fragoso et al. (A’); B, *Mawsonia gigas* (MCT 1370c-P) from the Berriasian of the Sanfranciscana Basin (redrawn from Carvalho & Maisey, 2008); C, *Mawsonia gigas* (FC-DPV 2977) from the Late Jurassic–Early Cretaceous Tacuarembó Fm., Uruguay (redrawn inverted from Toriño et al. 2021a); D, *Mawsonia gigas* (LPU 847) from the Tithonian of the Brejo Santo Fm, Brazil (redrawn from Batista et al., 2019); E, “*Mawsonia gigas*” or *Axelrodichthys* sp. (LPU 879) from the Tithonian of the Missão Velha / Brejo Santo Fm, Brazil (redrawn inverted from Batista et al., 2019); F, *Axelrodichthys lavocati* (unnumbered) from the ?Cenomanian Gara Sbaa Fm. (redrawn inverted from Tabaste, 1963); G, *Axelrodichthys araripensis*, (UERJ PMB 33) from the Aptian/Albian Santana Fm. (redrawn from Fragoso et al., 2018); H, *Axelrodichthys megadromos* (MDE F-61) from the terminal Cretaceous of Southern France (redrawn from Cavin et al., 2020).

*Description (Fig 2).* The maximum length of the angular is 208 mm and it maximal height is 70 mm, which corresponds approximately to a total body length of 1,50 m based one a reconstruction of a complete skeleton of a mawsoniid fish [38]. The angular is approximately triangular, with a slightly convex posterodorsal margin, a regularly concave anterodorsal margin and a ventral straight margin (Table 1 for measurements). The coronoid eminence, located mid-length of the bone, is rounded and slightly inclined forward. The labial (lateral) side is ornamented with a dense pattern of reticulated ridges oriented along the anteroposterior axis of the bone in its mid-depth, and oriented toward the coronoid eminence in the upper part of the bone. Along the ventral margin of the bone is a band without solid ornamentation, but with only faint ridges. The boundary between the two surfaces forms a small overhang, along which open oval foramina for the exit of the mandibular sensory canal. Four relatively small and irregularly arranged openings are visible (f.m.s.c). A depressed elongated smooth band extends along the half length of the anterodorsal margin of the bone. It corresponds to the articular surface for the dentary (ar.De). The labial (internal) face bears several structures. The coronoid eminence that contact the principal coronoid in *Mawsonia* and *Axelrodichthys* slightly protrudes inwards (sut.p.Co), but no clear sutural surface is visible. Ventral to the eminence is an arched depression starting posteriorly just below the eminence of the bone and widening anteriorly, with a well-developed ridge marking its ventral margin. This depression corresponds to the adductor fossa (a.f), which accommodated the adductor mandible muscles anchored on the ridge. On the posterior half of the bone, the main structure is a large ovoid raised sutural surface corresponding to the contact surface with the prearticular (con.Part). Dorsal and slightly anterior to this surface is another depression, here called the longitudinal fossa (l.f) which accommodated the articular and retroarticular bones. Below the raised ovoid sutural surface, runs a deep groove for the external mandibular branch of the VII nerve (gr.VII.m.ext). This groove fades anteriorly.

**Table 1:** *Mawsonia* sp., bone measurments (in mm).

	Angular 77688	Parasphenoid 77687	Basisphenoid 77728	Pterygoid 77686
Length	208	135 (max. preserved)	130	118 (max. preserved)
Height	70 (max)			
Width		73 (max. preserved)	91 (max. preserved)	116 (max. preserved)
Length of toothplate		112		+77
**Width of toothplate**		16		+13

*Identification (Fig 3).* This bone is identified as a coelacanth angular based on the pattern of the mandibular sensory canal and the openings from that canal, the path of the groove for the external mandibular ramus of the facial nerve and the presence of a ridge on the medial side which marks the floor of the adductor fossa. The specimen is referred with confidence to the *Mawsonia*–*Axelrodichthys* complex because of an ornamentation consisting of coarse ridges radiating, a slightly inflated lateral surface, the few oval openings of the sensory canal and the well-marked medial contact surface with the prearticular.

The angular bone is a comparatively common ossification of mawsoniid coelacanths due to its compactness and simple shape. Therefore, diagnostic characters have been repeatedly sought in this bone with the aim of separating genera and species within the *Mawsonia*—*Axelrodichthys* complex. Carvalho & Maisey [21], however, have drawn attention to the fact that "bones such as the angular and postparietal are known in several forms and have therefore tended to carry greater weight in systematic comparisons than they probably deserve”. Fig 3 illustrates a sample of angulars from specimens referred to various species of *Mawsonia* and *Axelrodichthys*. Maisey [101] and Forey [29] identified characters on the angular to distinguish the two genera, such as the deepest point of the bone positioned near the anterior margin in *Axelrodichthys*, while this point is located at mid-length in *Mawsonia*, and the overlap surface with the dentary short in *Axelrodichthys* and long in *Mawsonia*. The first of this character, the position of the deepest point, is well illustrated with the four examples of *Mawsonia gigas* (Fig 3A–3D) compared to the three species of *Axelrodichthys* (Fig 3E and 3F). The second character, the size of the overlap area, is not so obvious in the figure. This character is, anyway, related to the first character (position of the deepest point). The coronoid eminence with an anteriorly turned process is another characteristic distinguishing *Axelrodichthys* from *Mawsonia* according to several authors [29, 102]. In this regard, *’Mawsonia’ lavocati* (Fig 3F), from the mid-Cretaceous of North Africa, resembles *Axelrodichthys* more than *Mawsonia*, a generic reassessment previously obtained by Fragoso et al. [102] based on other characters not discussed here. The ornamentation is said to be stronger and mainly parallel to the longitudinal axis of the bone in *Mawsonia*, but weaker and radially arranged in *Axelrodichthys* [102]. We suggest that this difference mainly depends on the size of the specimen as shown by the large angular of *A*. *lavocati* figured by Tabaste [18] and reproduced here schematically (Fig 3F). This specimen shows a pattern more similar to large specimens of *Mawsonia*, although its outline is reminiscent to *Axelrodichthys*. The sutural contact surface with the main coronoid located at the highest point of the angular is small in *Mawsonia*, even absent in some specimens, and much more developed in *Axelrodichthys* [29, 103] (compare Fig 3A–3D and 3F–3H). Although never mentioned to our knowledge, it also appears that a contact surface with prearticular is present in most of the angulars of *Mawsonia* examined, although sometimes very small as in the specimen from Uruguay [24] (Fig 3C), while this surface seems to be absent in *Axelrodichthys*.

**Fig 4:**
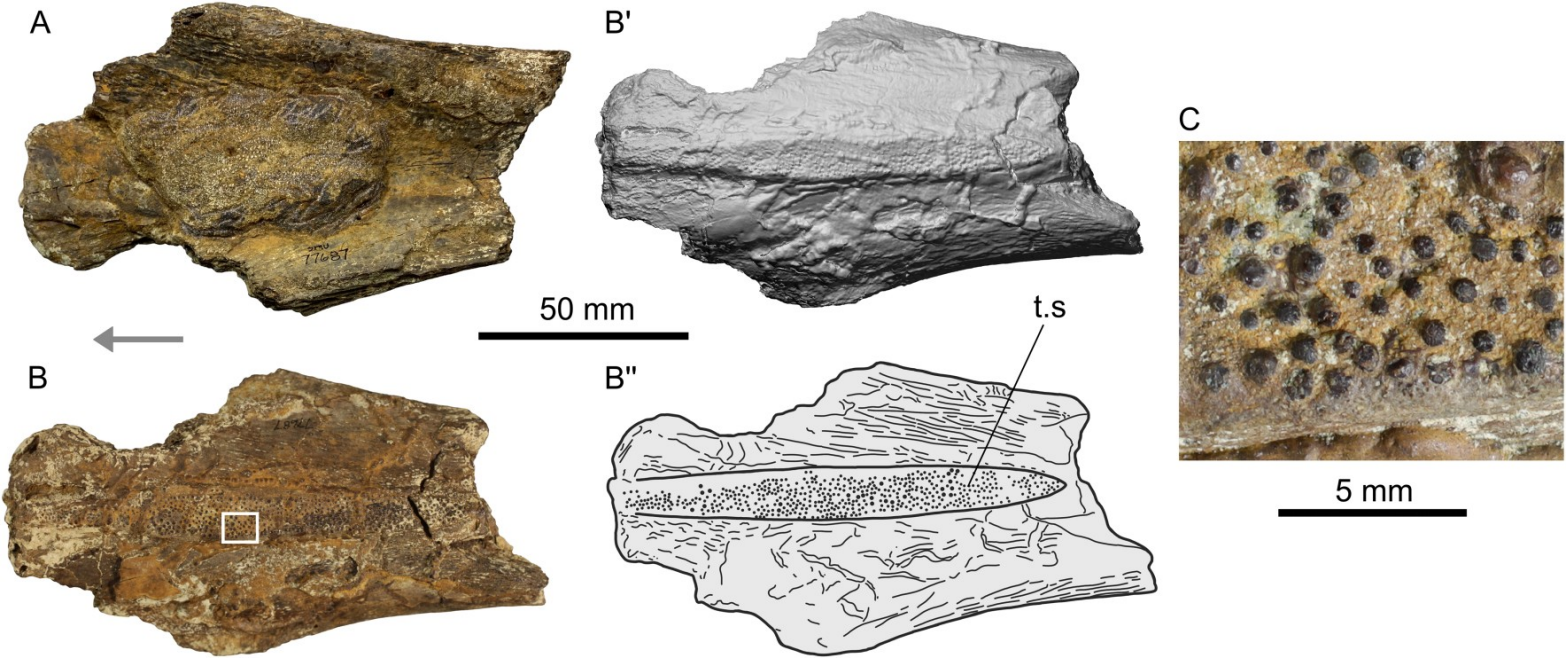
Photographs, surface rendering (apostrophe) and schematic drawing (double apostrophes) of anterior portion of parasphenoid (SMU 77687) of *Mawsonia* sp. from the Woodbine Formation, in dorsal (A) and ventral (B) views. The area indicated in B (white rectangle) is detailed in the microphotograph of C. Abbreviations: t.s, toothed surface. The arrow indicates the anterior part.

Subtle differences were also reported on angular in order to separate species within the genera *Mawsonia* and *Axelrodichthys*. Tabaste [18], for example, identified characters to distinguish “*M*.*” lavocati* from *M*. *libyca*, both from the mid-Cretaceous of North Africa, but other authors have regarded these differences as intraspecific variations [21, 29, 104]. Likewise, the angular shape of *M*. *brasiliensis* has been used by Yabumoto [105] in its diagnosis, e.g. posteriorly, the angular has a steep margin and is deep but narrow anteriorly, thus forming a more pronounced coronoid prominence than in other species [103]. But the shape of the coronoid eminence was found to be highly variable among a relatively large sample of angulars of *M*. *gigas* studied by Carvalho & Maisey [21], thus questioning the validity of the species *M*. *brasiliensis*, which is therefore considered by these authors as a synonym of *M*. *gigas*. *Axelrodichthys maiseyi*, from the Albian of the Codó Formation has a diagnosis based largely on characters of the angular, although a complete skull is known [106]. The validity of this species has been questioned by Fragoso et al. [102]. Finally, Cupello et al. [103] (Fig 2F and 2G) and Batista et al. [107] (Fig 4E–4H and Fig 3D and 3E) referred two angulars from the Tithonian Missão Velha / Brejo Santo Formation, northeastern Brazil to *M*. *gigas*. The two ossifications are well preserved and the ornamentation on their labial side consists of thin longitudinal ridges without radial pattern, slightly more marked in the larger specimen than in the small one (116 mm and 87 mm in length, respectively). The lingual side is, however, different between both specimens. The large specimen (Fig 3D, LPU 847) shows a rather shallow outline, with a very small or absent sutural contact area with the principal coronoid but with a well-developed contact area with the prearticular. The small specimen (Fig 3E, LPU 879) has a more pronounced coronoid eminence, a well-developed sutural contact surface with the principal coronoid but no visible contact surface with the prearticular. Batista et al. [107] considered that the differences between the two bones are due to ontogeny. The difference in size, however, is not very important between both angulars. Indeed, the larger specimen shows *Mawsonia* characters, while the smaller one is more reminiscent of *Axelrodichthys*, except for the position of the coronoid eminence located approximately at mid-length of the bone. More material is pending to confirm or reject this hypothesis, but if the identification of *Axelrodichthys* is confirmed in the Brejo Santo Formation, this occurrence would represent the oldest record for this genus.

Based on this brief review of the angular condition within the *Mawsonia*—*Axelrodichthys* complex, the systematic assignment of SMU 77688 is somewhat equivocal. Based on the position of the coronoid eminence, it is reminiscent of *Mawsonia* because this eminence is located approximately mid-length of the bone. But the form of the eminence is somewhat intermediate between *Mawsonia* and *Axelrodichthys*, with the process slightly more hooked than in most specimens of *Mawsonia* (although in *M*. *gigas* from the type locality (Fig 3A) has a well-marked process), but less protruding than in most instances of *Axelrodichthys*. The absence of, or poorly developed sutural contact with the principal coronoid at the tip of the eminence is also reminiscent of *Mawsonia*. Finally, the presence of a large ovoid contact surface with the prearticular is a newly recognized trait that appears only in *Mawsonia*. The angular characters previously used to distinguish species are too ill-defined so far to allow the assignment of SMU 77688 to a known species, or to recognize it as a new species. Therefore, the angular is referred to *Mawsonia* sp. for the time being.

#### Parasphenoid (SMU 77687)

*Description (Fig 4).* The parapshenoid is represented by a robust fragment including the almost complete toothed area, therefore suggesting an anterior portion of the bone (Table 1 for measurements). Its size suggests a much larger individual than the one corresponding to the angular. The bone is highly concave in dorsal view, with a nodule of sediment obscuring some of its surface features (Fig 4A). In ventral view the bone is convex, and the toothed surface constitutes a flat and raised plate, anteroposteriorly elongated, which extends throughout most of the preserved portion of the bone (Fig 4B–B”). Considering the width and robustness of the bone, this plate appears as notoriously narrow and short. Both ends of this plate seem to be particularly sharp. The best preserved end of the plate is located where the fragment becomes narrower (Fig 4B–B”, right end). The opposite end of the fragment is less well preserved, but it seems to have originally been wider, possibly representing the anterior part of the bone close to where the lateral ethmoids were attached (Fig 4B–B”, left end).

The teeth are sparse throughout the plate and have variable sizes. They are low and rounded, radially striated and with darker and brighter surfaces than bone, possibly preserving their enamel cover (Fig 4C).

*Identification*. Due to the state of preservation of the specimen, the configuration of the teeth is one of the few features that could be used to approach a general taxonomic identification. The morphology of the teeth (see above) matches with the reported for the derived mawsoniids *Axelrodichthys* [27–29, 102] and *Mawsonia* [24, 103], although it must be warned that the presence of striations is not exclusive to members of this family [29]. As a note, although the dentition of the marine mawsoniid *Trachymetopon* (closely related to the previous two) has not been described until now, a pterygoid from a giant specimen coming from the Upper Jurassic of Normandy [38, 108] also bears small radially striated low denticles (L.C. pers. obs.).

The contour of the toothed plate seems to be variable in *Axelrodichthys* and *Mawsonia*, although no complete parasphenoids with well-preserved toothed plates are currently referred to the latter. As an example, in the problematic species *‘Mawsonia’ lavocati* the posterior end varies from highly pointed to rounded [19, 27, 109]. It must be noted that this species has been recently transferred to *Axelrodichthys* [102]. Therefore, the contour of the toothed plate in SMU 77687 cannot be taken as conclusive.

On the other hand, the referred tooth pattern separates it from that known for another giant coelacanth: The marine *Megalocoelacanthus* (Latimeriidae), in which the teeth are villiform and no striae are reported [110]. Moreover, in *Megalocoelacanthus* the parasphenoid seems to be considerably deeper than suggested by SMU 77687, the latter resembling more to *Mawsonia* and *Axelrodichthys* in this regard.

Finally, the large size and robustness of the specimen is more consistent with previous giant *Mawsonia* records, than with *Axelrodichthys* (*e*.*g*. [101]). Considering this and the arguments presented in the previous section regarding the assignment of the angular, SMU 77687 is also tentatively referred to *Mawsonia* sp.

#### Basisphenoid (SMU 77728)

**Fig 5:**
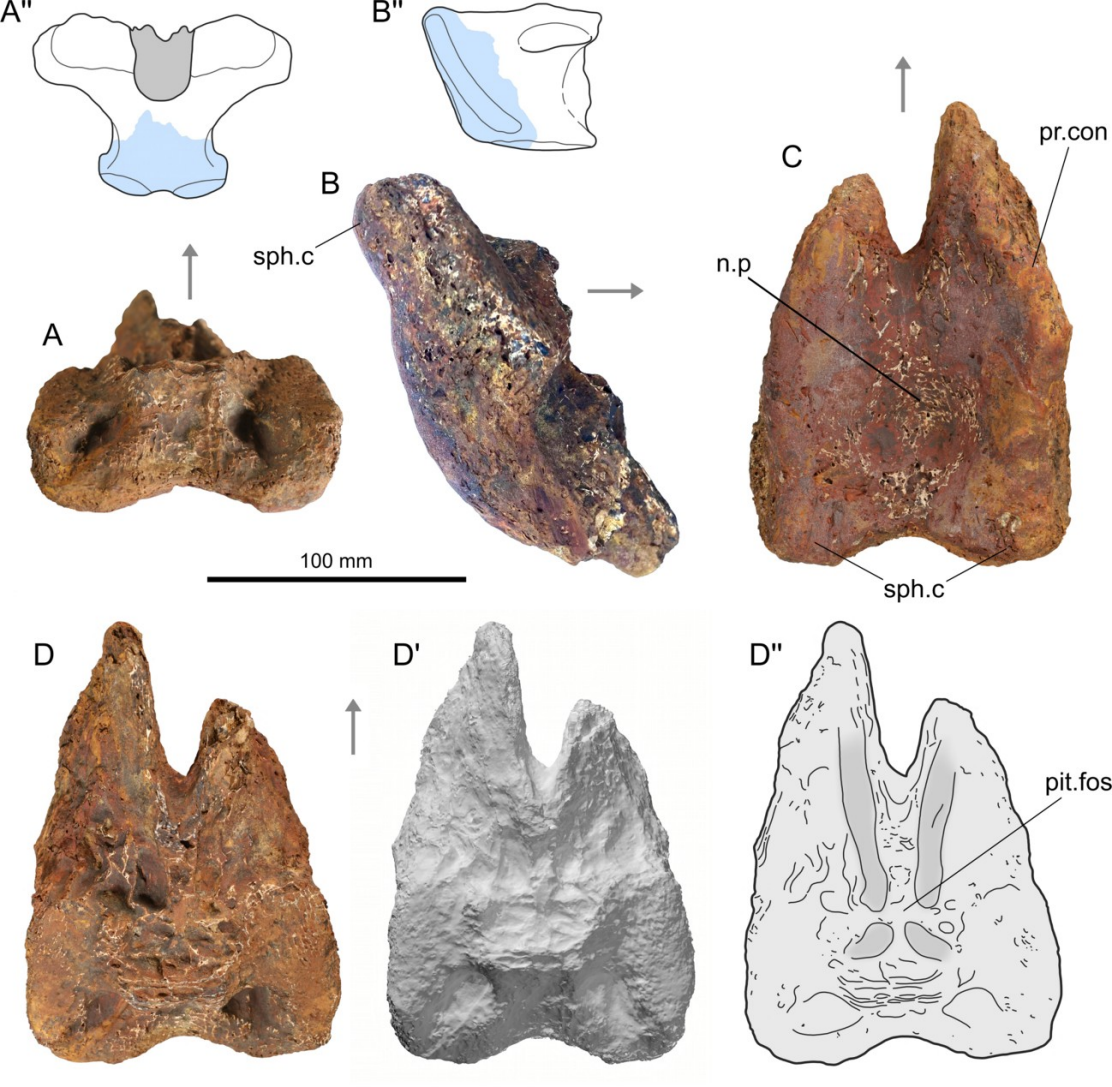
Photographs, surface rendering (apostrophe) and schematic drawings (double apostrophes) of a posterior portion of basisphenoid (SMU 77728) of *Mawsonia* sp. from the Woodbine Formation, in dorsal (A), right lateral (B), posteroventral (C) and anterodorsal (D) views (A’ and B’, redrawn from Wenz, 1981). Abbreviations: n.p, notochordal pit; pit.fos, pituitary fossa; pr.con, processus connectens; sph.c, sphenoid condyles. The arrows indicate the anterior part.

*Description (Fig 5).* SMU 77728 represents a posterior fragment of a large basisphenoid preserving just the posteroventral surface of the bone (Table 1 for measurements). The rough texture of the piece suggests certain degree of erosion, which is particularly notable in dorsal and anterior views (Fig 5A and 5D). The *processus connectens* are poorly preserved, and just part of the left one can be observed (Fig 5C). The sphenoid condyles are partially eroded, being recognizable in posteroventral view (Fig 5C). As a note, a paired excavation can be seen at the dorsal portion of the inner surface of the piece (Fig 5A and 5D). It is considered that these depressions can be due to poor preservation (i.e. a differential erosion of the endochondral bone). A central depression can be observed in posteroventral view, which is interpreted as the notochordal pit (Fig 5C).

The specimen measures 130 mm at is maximum length and 91 mm at its maximum width. Its dimensions indicate a giant individual, with a larger size compared to that of the corresponding to the parasphenoid. The estimate based on a reconstruction of mawsoniid provided in [38] indicates a total body length of about 4 meters.

Due to its topological situation, the inner surface of the piece represents the floor of the pituitary fossa of the basisphenoid (Fig 5D–D”). Besides its partially eroded surface, it still contains some particular features possibly related to the anatomy of the pituitary gland at this point, particularly a posterior pair of two small cavities and an anterior pair of two elongated depressions (marked with soft shadows in Fig 5D”). The latter constitutes a singular arrangement, considering the topological changes of the gland during ontogeny, as known for *Latimeria* [111]. Unlike documented for the early stages of the living coelacanth, in the adult the pituitary fossa is only occupied by the anteriormost projection of the gland (the hypophyseal duct). Thus, it would be expected a simple configuration of the floor of the pituitary fossa at the adult stage. As a note, these features have been recently used in the fossil record of coelacanths to the identification of juveniles’ basisphenoids in the genus *Trachymetopon* [38].

The case of SMU 77728, a basisphenoid with a large size that could match for an adult but with the presence of cavities at its inner surface, constitutes a singularity that will deserve a deeper comparative study on the basis of better preserved specimens.

*Identification*. The state of preservation of the bone prevents an accurate identification. For example, it is considered that the poor preservation of the sphenoid condyles prevents its use to discriminate between mawsoniids or a latimeriids, following the arguments proposed by authors [112, 113]. Nevertheless, among giant coelacanths, the posteroventral face of the basisphenoid of *Megalocoelacanthus* (latimeriid) seems to be more transversely narrow and rostrocaudally elongate than in *Trachymetopon* and *Mawsonia* (mawsoniids) [38, 101, 110, 114], and these differences are even more pronounced in the latter genus. In this regard, the proportions of SMU 77728 are closer to *Mawsonia* than to the other cited giant genera. It is therefore temporarily referred to *Mawsonia* sp., although we do not exclude the possibility that this ossification belongs to *Axelrodichthys*, or even another genus, keeping in mind that several genera of coelacanths can live in sympatry as illustrated with the Aptian/Albian Santana Formation, Brazil [101].

#### Pterygoid (SMU 77686)

**Fig 6:**
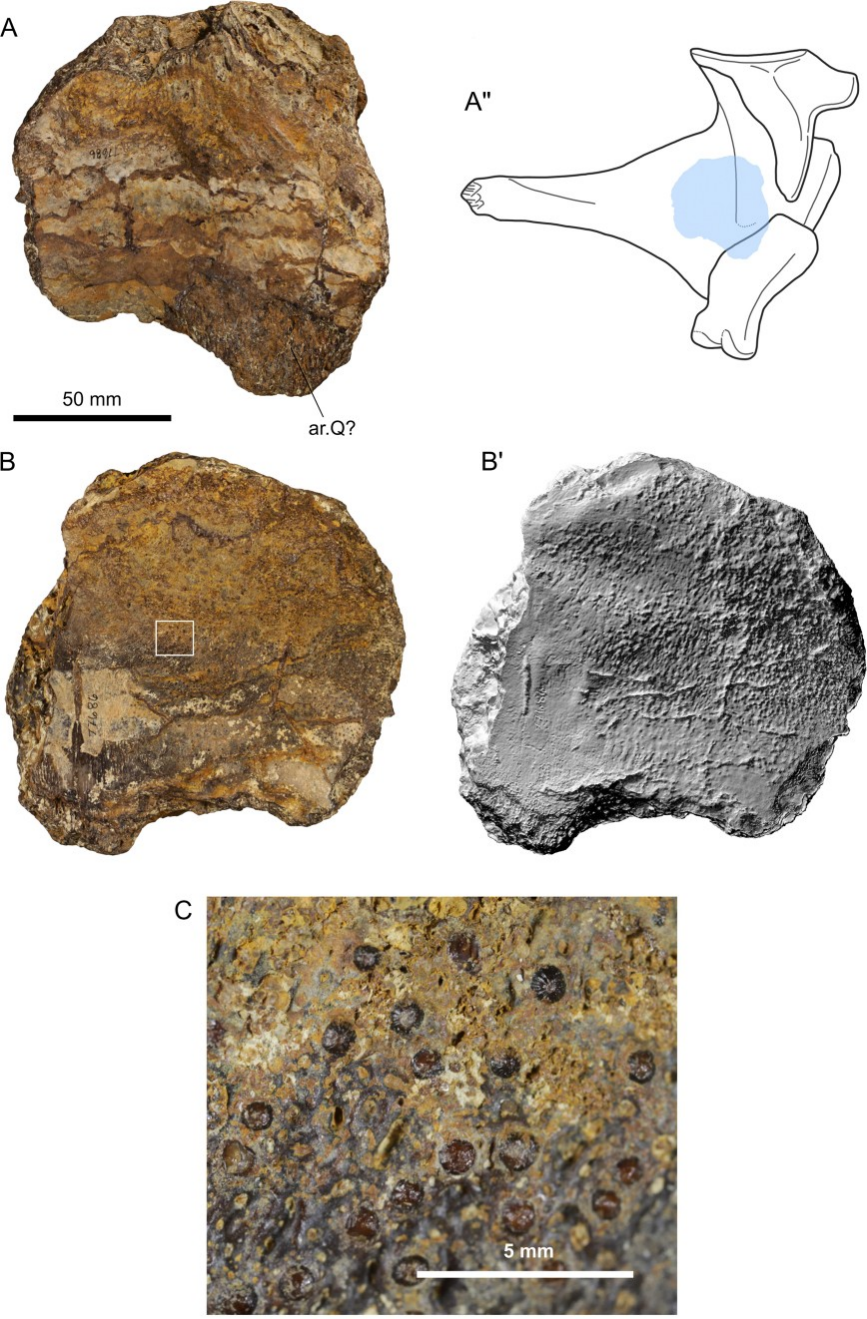
Photographs of a portion of pterygoid (SMU 77686) of *Mawsonia* sp. from the Woodbine Formation, in lateral (A) and medial (B) views, surface rendering (apostrophe) and schematic drawing (double apostrophes) of a left palatoquadrate in lateral view, indicating the tentative position of the piece (redrawn from Dutel et al. 2014). The area indicated in B (white rectangle) is detailed in the microphotograph of C. Abbreviation: ar.Q, articular surface for quadrate.

*Description (Fig 6).* This is a massive, flat and thick element belonging to a large bone, measuring 138mm at its maximum length (Table 1 for measurements). A large area of one of its surfaces is covered with small striated teeth, with the same constitution than those of the parasphenoid fragment (SMU 77687; see above) (Fig 6B and 6C). Due to its heavy constitution and wide toothed surface, it is interpreted as a portion of a big pterygoid, instead of a paired toothed bone from the lower jaw (e.g. prearticular, coronoids) or branchial apparatus, which tend to be relatively more gracile and thin. The thickness of the piece and the possible presence of an articular face in the non-toothed surface suggest this could correspond to the posterior part of a left pterygoid (Fig 6A–A”).

*Identification*. As for the parasphenoid, the state of preservation of the bone also prevents an accurate identification. Only a few considerations can be stated on the basis of the size, robustness and dentition, as already detailed for the parasphenoid (see above). Therefore, the specimen is tentatively referred to *Mawsonia* sp., with the same limitation raised above for the identification of the basisphenoid.

### Ray-finned and coelacanth genera survivorships (Fig 7)

**Fig 7:**
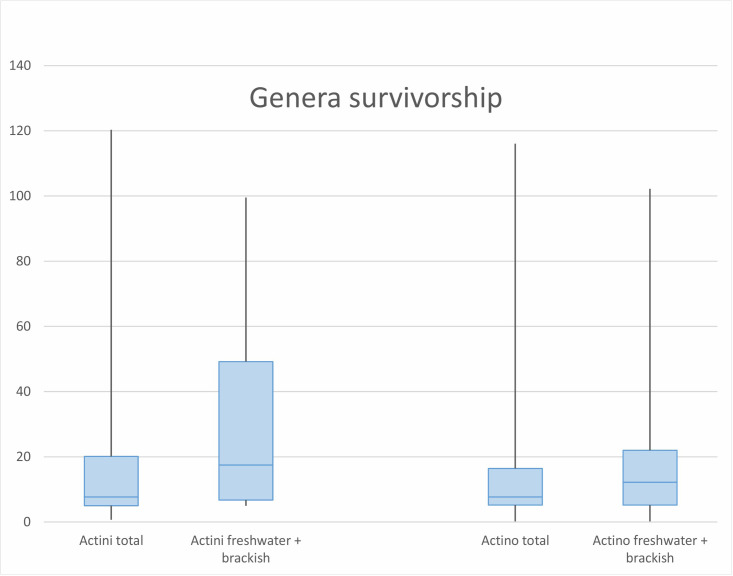
Comparison of genera survivorship in myrs between coelacanths (left) and ray-finned fish (right), distinguished between paleoenvironments. Abbreviations: Actini, Actinistia (coelacanths); Actino, Actinopterygii (ray-finned fishes).

On the basis of the database mentioned in the Material and Methods section, we calculated average time durations for distinct clades and paleoenvironments. The average time duration of the ray-finned fish genera from the Devonian to the Palaeocene is 13.3 million years (myrs) all environments combined, 11.1 myrs for marine genera only, 14 myrs for strictly freshwater genera and 17.2 myrs for freshwater and mixed genera. For coelacanths, these values are 19.5 myrs, 15 myrs, 17.9 myrs and 31.3 myrs respectively, i.e. between 4 and 6 million years longer than for the ray-finned fishes depending of the environment, except for freshwater and mixed genera combined which are more than 14 myrs longer on average.

Of the 1108 time ranges calculated for the ray-finned genera, only 24 have a longer range than *Mawsonia* and *Axelrodichthys*. Interestingly, ten of them have living species and six probably correspond to the "seven genera of ganoid fish" cited by Darwin [115] as examples of "living fossils" when he first coined this expression in *The Origin of Species* (*Lepisosteus*, *Atractosteus*, *Amia*, *Polypterus*, *Acipenser*, *Polyodon*). Of the 58 calculated time ranges of the coelacanth genera, four have longer intervals than *Axelrodichthys* and *Mawsonia*. All four (*Macropoma*, *Holophagus*, *Rhabdoderma*, *Coelacanthus*) are possibly waste basket taxa that deserve systematic revisions [29]. They may include too many species and, therefore, inflate time ranges. However, uncertainties in the demarcation of species within these genera also reflect the lack of clear morphological differences indicating slow morphological evolution within these lineages.

## Discussion

### Biogeography (Fig 8)

**Fig 8:**
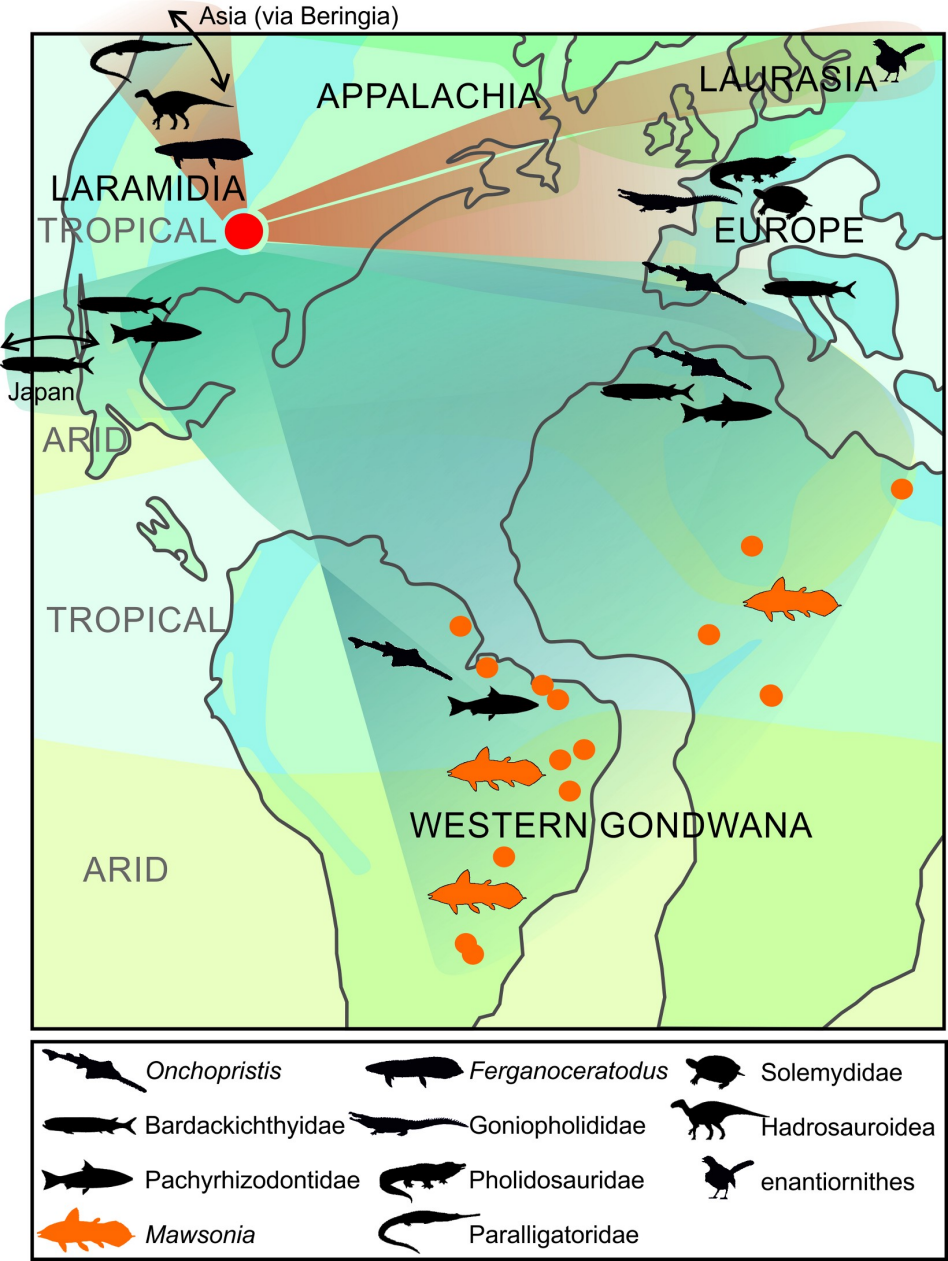
‘Mid’-Cretaceous paleogeographical map of western Laurasia and western Gondwana showing biogeographical affinities of vertebrates from the woodbine formation (red circle). Brown areas indicates continental vertebrates and the blue areas indicate brackish and marine vertebrates. Orange dots indicate approximate locations of records of *Mawsonia*.

#### Signal from the vertebrate assemblage

The Woodbine vertebrate assemblage represents one of the oldest known Appalachian continental assemblages, and its components are important in reconstructing Appalachian paleogeographic connections with other landmasses. In particular, the Arlington Archosaur site (AAS) has produced a wide range of diverse and well-preserved vertebrates [116] whose biogeographic affinities are described below, together with vertebrates from other localities.

The turtle assemblage from the Woodbine Formation is distinct from the Laramidian assemblage of similar age [85]. The occurrence of the solemydid *Naomichelys* indicates European affinities since this Late Jurassic to Late Cretaceous family of turtles is restricted to North America and Europe [117]. Similarly, the occurrence of the “*Trinitichelys*”, which belongs to the endemic North American baenids, indicates Laurasian affinities since the closest relatives, the paracryptodires, do not occur in Gondwana [85, 118]. The crocodyliform *Woodbinesuchus*, *Terminonaris*, *Deltasuchus motherali*, *Scolomastax sahlsteini* [90] are known from the AAS. The biogeographical signal of *Deltasuchus* is not very informative because it, together with its sister genera *Paluxysuchus* from the Early Cretaceous of North America, are sister to a large clade gathering goniopholidids plus eusuchians [88]. *Woodbinesuchus* is a goniopholidid, a family restricted to Laurasia [87] and *Terminonaris* is a pholidosaurid showing clear European connections [89]. *Scolomastax sahlsteini* has affinities with Asian paralligatorid genera, which would indicate interchanges across the Beringian land bridge in the Early Cretaceous before the completion of the Western Interior Seaway [90]. The enanthiornithin bird *Flexomornis howei* has Laurasian affinities [96], while the hadrosauroid dinosaur *Protohadros byrdi* [93], together with the coeval *Eolambia* from Utah clearly display Asian affinities [96].

The Woodbine Formation fish assemblage shows a less straightforward paleogeographic signal [77]. In their overview of the Cretaceous shark assemblages from Texas, however, Cappetta & Case [78] noted that the Cenomanian assemblage is the one with strongest affinities with other parts of the world, notably with Europe and North Africa. Among these shark taxa are *Haimirichia* (‘*Carcharias*’) *amonensis* known in the upper Albian and Cenomanian from USA, Europe and North Africa, with few occurrence in Angola, Lebanon and Japan [119]. Dunkle [73] referred a fragmentary tooth without the apex preserved to the sclerorhynchid shark *Onchopristis* cf. *numidus*, an evidence of North African affinities since *O*. *numidus* is very widespread in the mid-Cretaceous of North Africa [80]. McNulty & Slaughter [74] described from the Woodbine Fm. three more complete teeth showing two cusplets, instead of one in *O*. *numidus*, and coined the species *O*. *dunklei* for the Texan taxon. This species was then recorded in Spain and Western France [78, 120]. Martill & Ibrahim [121] recorded in the Cenomanian Kem Kem Group in Morocco the presence of some rostral teeth with two cusplets. They considered that these rare teeth are probably abnormal forms of *O*. *numidus*, although they do not exclude the possibility that 1) two sympatric species occurred in North Africa, with *O*. *dunklei* being very rare, or 2) the occurrence of a single species with morphological variation on a wide geographic range extending across the Atlanto-Tethyan province. Pereira and Medeiros [122] described *Atlanticopristis equatorialis*, a sclerorhynchid from the Cenomanian Formation of Alcântara, Brazil, which differs from *Onchopristis* by bearing several cusplets on its two cutting edges, but sharing with *O*. *dunklei* the presence of several cusplets on the posterior edge. In a recent study, Villalobos et al. [120] showed that the main difference between the rostral teeth of *O*. *numidus* and *O*. *dunklei* is that the orthodentine-filled cap is thicker and the pulp cavity is smaller in the first species than in the second. Whatever the systematic position of *O*. *dunklei*, its presence indicates European and Gondwanian affinities, in particular with the Alcântara Formation in South America and the Kem Kem Group in Africa which also yielded remains of *Mawsonia*.

*Ceratodus carteri* is a lungfish described by Main et al. [83] based on a small sample of upper and lower tooth plates. Tooth plates of the genus *Ceratodus sensu stricto* have robust ridges, and have a crushing flat surface that does not match the pattern seen in *C*. *carteri*. The presence of five and four ridges in the upper and lower dental plates, respectively, the internal angle of about 90° and the first notch slightly deeper than the following ones in the upper and lower dental plates are reminiscent of the dental plates of Asian species of *Ferganoceratodus* [123]. We suggest that *C*. *carteri* should be included in the genus *Ferganoceratodus*, as well as the specimen referenced by Harrell & Ehert [124] from the Santonian Eutaw Formation also located in the Appalachians in the Late Cretaceous. To our knowledge, there is no convincing occurrence of *Ferganoceratodus* in the mainland masses of Gondwana. If this identification is confirmed, these occurrences of *Ferganoceratodus* represent further evidence of dispersal from Asia, where this genus is known in the Jurassic and Lower Cretaceous to North America where it persists until the Upper Cretaceous.

Recently, two stem teleosts have been described in the Woodbine Formation and the overlying Tarrant Formation. Hacker & Shimada [82] recorded an ichthyodectiform, *Bardackichthys carteri*, which they included in a new family called Bardackichthyidae which comprises several species of *Hackelichthys* and *Amakusaichthys*. The first genus occurred in the Cenomanian—Albian of Mexico [125], Morocco and Europe [126], while the second was present in the Santonian of Japan [127], all from marine deposits. The second stem teleost is a pachyrhizodontid from the Tarrant Formation, *Polcynichthys lloydhilli* [84], whose closest relatives are *Goulmimichthys*, from the Turonian of Mexico and Morocco, and *Rhacolepis*, from marine and non-marine deposits of the Lower Cretaceous of South America.

Overall, the continental component (freshwater and terrestrial) of the Woodbine Formation vertebrate assemblage shows mainly Laurasian affinities, with the exception of *Onchopristis* present in Western Gondwana, while the marine component made of stem teleosts shows Tethyan and Pacific affinities with rare Western Gondwanan affinities (*Rhacolepis* and *Goulmimichthys*).

#### Signal from *Mawsonia*

**Fig 9:**
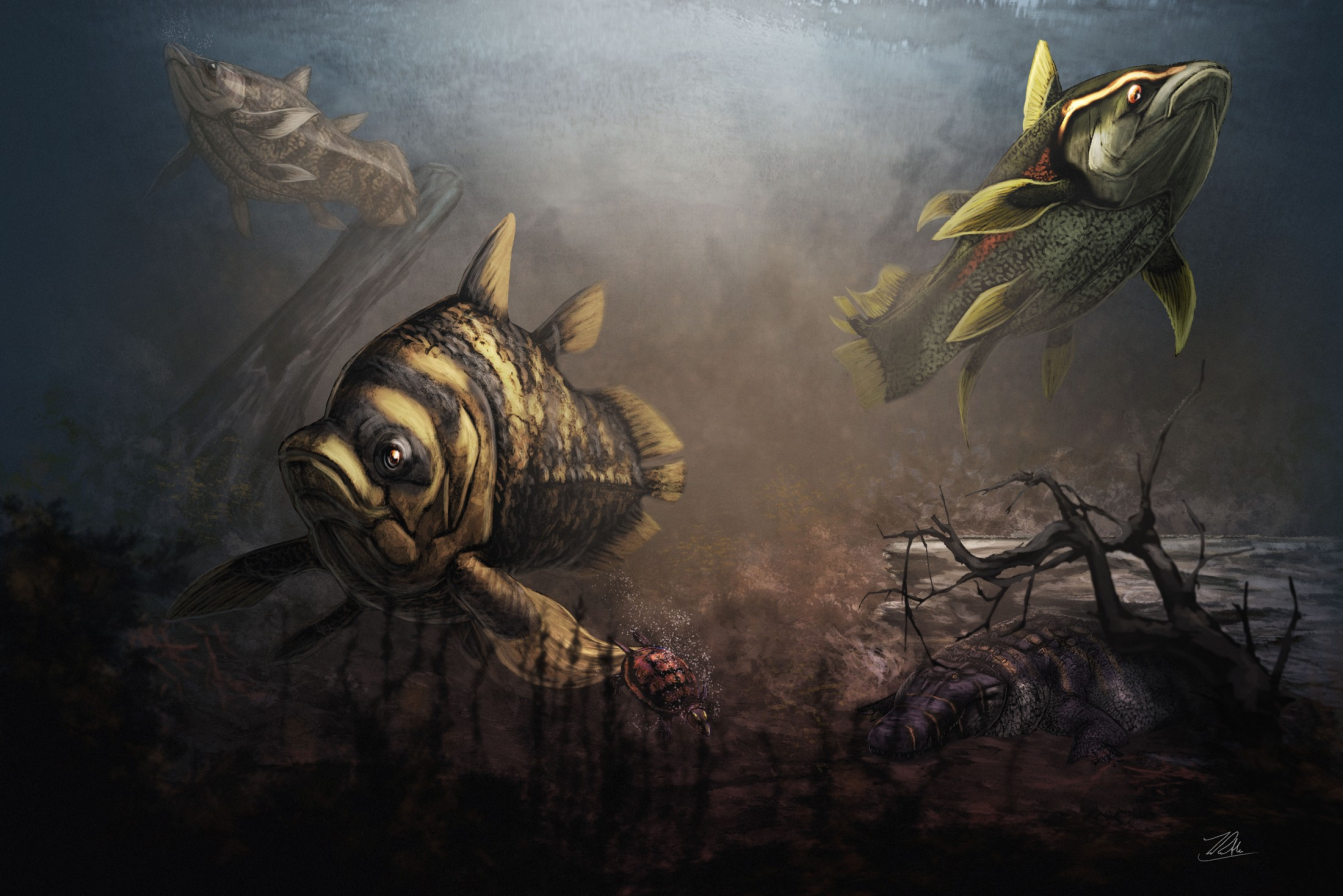
Reconstruction of *Mawsonia* sp. roaming in the brackish or fresh water costal environment of Texas during the Cenomanian. Original artwork by Zubin Erik Dutta.

The Texan occurrence of *Mawsonia* originates from a continental and coastal environment (Fig 9), as is the case for most occurrences of *Mawsonia* / *Axelrodichthys*. In fact, the only marine paleoenvironment that produced these genera is that of the Aptian/Albian Santana Formation, Brazil, which was probably a closed marine environment with strong variations in salinity and episodes of freshwater inflows, but not connected offshore. An occurrence of *Axelrodichthys* cf. *A*. *araripensis* has been reported from the marine Albian Tlayúa quarry in Mexico [128], but unfortunately the specimen has not been described and is now lost [129] (K. González-Rodríguez, personal communication, September 2021). If this occurrence is confirmed, it means that the Texan find is not the only Cretaceous occurrence of a mawsoniid in North America. With the exception of this uncertain Mexican marine occurrence, the only other exception of *Mawsonia* / *Axelrodichthys* fossil has ever been found in open sea sediments is an isolated ossified lung recently discovered in the Late Maastrichthian phosphate deposits in Morocco, which represents the last known fossil record of a coelacanth [23]. This interesting find may indicate a return of mawsoniids to the marine environment, although it cannot be excluded that this fragment of a corpse was transported from a river system, as it was the case with continental animals found in the phosphate basins, such as dinosaurs [130–132]. We consider that *Mawsonia* and *Axelrodichthys* were mainly freshwater inhabitants with brackish abilities, but without the ability for long marine dispersals. The geographical distribution of *Mawsonia* and *Axelrodichthys* has been explained by vicariant events between South America and Africa, and by continental or coastal dispersions, but also across short sea barriers, as suggested for the Malagasy and European occurrences [133]. The North American occurrence, however, involves rethinking biogeographic models, but this can only be done correctly once the phylogenetic relationships of the *Mawsonia* / *Axelrodichthys* complex will be resolved. We can even hypothesize that the Cretaceous distribution of the genera pair is the result of an ancient Pangean distribution and experienced the breakup of the supercontinent. In this case, the closely related Jurassic marine genus *Trachymetopon* [30, 38, 108, 112, 134] should be included in the scenario. But again, a well-supported phylogeny is needed to draw biogeographic models.

#### Comparison between *Latimeria* and *Mawsonia* (Fig 10)

Although *Latimeria* is a marine fish and *Mawsonia* was a freshwater to brackish fish, they share evolutionary traits. The long-range duration of *Latimeria* is based on genetic evidence that indicates a split between the two extant and morphologically close species occurring 30 to 40 million years ago [3]. *Mawsonia*’s stratigraphic range, as documented by its fossil record, is still uncertain based on the attribution of some of the recovered fossils. It covers a range of at least 50 million years, from the Upper Jurassic to the early Late Cretaceous with the Texan find described here, or perhaps nearly 85 million years if we refer the Maastrichtian Moroccan occurrence to this genus. Likewise, the stratigraphic range of *Axelrodichthys* covers a range of nearly 50 million years, from the late Early Cretaceous to the terminal Cretaceous, or nearly 85 million years if the Late Jurassic occurrence from the Missão Velha / Brejo Santo Fm. discussed above refers to this genus.

**Fig 10:**
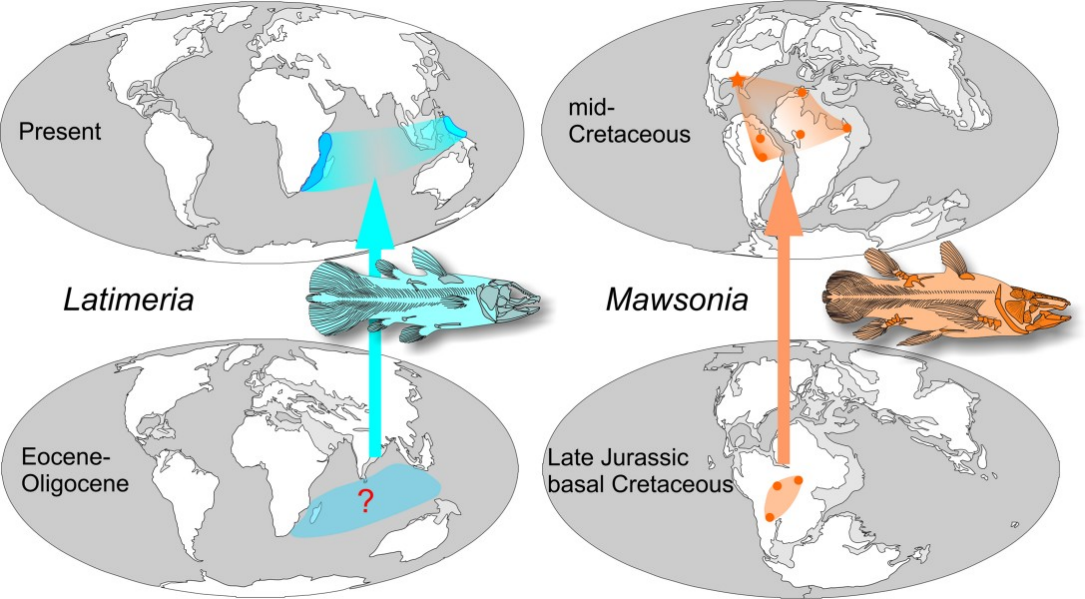
Comparison of the geographical distributions of the two extant *Latimeria* species and their supposedly common ancestor 30 to 40 million years ago (left) with those of *Mawsonia* in the mid-cretaceous, about 100 million years ago and in the Late Jurassic–basal Cretaceous, about 145 million years ago (right).

The comparison between the paleobiogeographic history of *Latimeria* and *Mawsonia* over their long stratigraphic range is instructive (Fig 10). While a vicariant model at the origin of the distribution of *Latimeria* remains hypothetical, i.e. a restricted original distribution of the common ancestor of the two species which enlarged during the opening of the Indian Ocean and was affected by the India’s northward displacement, a probable vicariant model is brought to light for *Mawsonia* on the basis of its fossil record. In the Late Jurassic—basal Cretaceous, the geographical range was limited to the center of Western Gondwana. Then, in the middle of the Cretaceous, the range expanded and covers the entire northern part of Africa and South America, with an extension demonstrated here as far as North America. However, it is still difficult to know if this pattern is the result of a vicariance or of a succession of dispersal events, but the distribution corresponding to an initial cradle widening peripherally over time favors the first hypothesis (Fig 10 right).

The Texan discovery dramatically expands *Mawsonia*’s geographic range and is further evidence that there is a link between geographic distribution and survival. This is a clue that the situation was probably similar for the *Latimeria* lineage.

## Conclusion

The Texan discovery of *Mawsonia* sp. adds an important new component to the Woodbine vertebrate fauna. It is an unexpected Gondwanian representative in this Appalachian assemblage with predominantly Laurasian (European and Asian) affinities. It considerably increases the geographical distribution of this genus, and confirms its occurrence at the beginning of the Late Cretaceous.

This discovery also has implications for a general feature of animal evolution. "Living fossils" is an ill-defined concept first used by Darwin [115] to characterize organisms that share, among other characteristics, a slow rate of evolution. When the fossil record is considered, this trait corresponds to long stratigraphic extensions of taxonomic units. In this sense, *Mawsonia*, based on its fossil record and *Latimeria*, based on genetic and morphological evidence, are “living fossils” if we consider their very long genus survivorship. The two genera also have a very large geographical distribution. Some of the rare Actinopterygians with a longer range than *Mawsonia*, and already considered "living fossils" by Darwin [115] such as *Lepisosteus*, *Atractosteus* and *Amia* also had a much larger distribution in the past than their present restriction to North and Central America. It should be noted, however, that a wide geographical distribution is not considered a characteristic of "living fossils" by Darwin in an old reference to this concept which appears in a letter to JD Hooker dated December 24, 1858. For Darwin, at conversely, the most derived forms ("improved forms") are those which inhabit the largest areas [135]:

*It is that species inhabiting a very large area*, *& therefore existing in large numbers & which have been subjected to the severest competition with many other forms*, *will have arrived through natural selection*, *at a higher stage of perfection than the inhabitants of a small area*.*—Thus I explain the fact of so many anomalous or what may be called “living fossils” inhabiting now only fresh-water*, *having been beaten out & exterminated in the sea by more improved forms; thus all existing Ganoid fishes are fresh-water as is Lepidosiren & Ornithorhynchus &c*.*—*[135]

The slow morphological evolutionary rate observed during most of the history of coelacanths may have two main causes: endogenous or environmental. *Latimeria*’s supposedly constant mesobenthic environment has been suggested as the reason for its slow morphological transformation [136], but the case of *Mawsonia* illustrated here clearly shows that a similar slow evolution occurred in very different environments, i.e. fresh and brackish waters. Therefore, we favour here the presence of biological rather than environmental characteristics specific to the *Latimeria* and *Mawsonia* lineages. The recent discovery by Mahé et al. [137] that *Latimeria* has an advanced age of sexual maturity, perhaps around 50 years old, and a very long gestation period, of around 5 years, is a potential parameter that explains the slow evolution of its lineage. However, we note that slow morphological evolution is not a constant rule in latimeroids (latimeriids plus mawsoniids) as illustrated by certain Middle Triassic latimeriid fish morphologically very divergent from the general coelacanth Bauplan [138, 139].

The link between large geographic distribution and extinction resilience demonstrated here for *Mawsonia* is a clue that a similar situation exists for *Latimeria*, which allowed this genus to live for tens of millions of years. But it would be an inestimable loss if this survival force of this unique representative of a 420 million year old lineage were wiped out through human activities.
